# Supplementary material for: Differentiating between Lymphoma and Metastasis Presenting as Solid Cerebellar Mass Lacking Necrosis
Source: Diagnostics (Basel). 2024 Oct 6;14(19):2228. doi: 10.3390/diagnostics14192228 (PMC11482554; doi:10.3390/diagnostics14192228)
Supplement: Supplementary file 1 [file diagnostics-14-02228-s001.zip › diagnostics-3168608-supplementary.pdf]

Table S1. Primary central nervous system lymphoma group.

Patient No.	Age/Sex	Tumor size, mm	Enhancement pattern	Surface involvement	Streak-like edema	Serrate sign (planes)	Branch-like enhancement	Outward spikes	Section thickness of contrast enhanced T1WI(AxCxS), mm	Pathologic procedure
1	52/M	1.8	Homogeneous	X	O	X	X	X	5x5x5	Supratentorial Bx.
2	72/F	1.5	Homogeneous	X	O	X	X	X	5x5xNA	Tumor removal
3	81/F	1.5	Homogeneous	X	O	X	X	X	4x4x4	Supratentorial Bx.
4	63/M	4	Homogeneous	O	O	(C, S)	X	O	7x7x7	Partial tumor removal
5	63/F	3.5	Homogeneous	X	O	(A, S)	O	X	5xNAx5	Craniotomy Bx.
6	63/M	4.6	Homogeneous	X	O	(C, S)	O	X	5x5x3	Burr hole Bx.
7	64/F	2.4	Homogeneous	X	X	(C, S)	O	X	4x4x1	Burr hole Bx.
8	73/M	4.1	Heterogeneous	O	O	(A, C, S)	O	X	5x5.5x5.3	Burr hole Bx.
9	58/M	2	Homogeneous	X	O	(C)	O	O	5x5x5	Burr hole Bx.
10	56/M	1.9	Homogeneous	X	X	(A, S)	O	X	5x5x1.5	Supratentorial Bx.
11	53/F	3.2	Homogeneous	O	O	X	X	X	5x5.5x5.3	Partial tumor removal
12	58/M	1.9	Homogeneous	X	O	X	X	X	5x5x1.5	Burr hole Bx.
13	76/M	2.4	Homogeneous	X	O	(A, S)	O	X	5x5x2.6	Burr hole Bx.
14	76/M	2.3	Homogeneous	X	O	(A, C, S)	O	X	4x4x1	Supratentorial Bx.
15	68/M	4.5	Heterogeneous	O	O	(C, S)	O	X	5x5.5x5	Burr hole Bx.
16	60/F	3	Homogeneous	X	O	(A, C, S)	O	X	5x5x1.5	Burr hole Bx.
17	72/M	2.8	Homogeneous	X	O	(S)	X	O	4x4x1	Burr hole Bx.
18	74/F	2.8	Homogeneous	X	O	(S)	X	O	5x5x2	Burr hole Bx.
19	57/M	3.4	Homogeneous	O	O	(S)	X	O	4x4x1	Supratentorial Bx.
20	58/M	1.8	Homogeneous	X	X	(A, C, S)	O	X	4x4x1	Supratentorial Bx.
21	37/M	1.8	Homogeneous	O	O	X	X	X	4x4x1	Burr hole Bx.

22	63/M	1.9	Homogeneous	X	O	(A, C, S)	O	X	5x5x1.5	Supratentorial Bx.
23	79/M	2.9	Homogeneous	O	O	(S)	O	X	5x5x5	Supratentorial Bx.
24	61/M	2.7	Homogeneous	X	X	(A, C, S)	O	X	5x3x1	Burr hole Bx.

A, Axial; C, Coronal; S, Sagittal; Bx., Biopsy, NA; Not available

Table S2. Metastasis group.

Patient No.	Age/Sex	Tumor size, mm	Enhancement pattern	Surface involvement	Streak-like edema	Section thickness of contrast enhanced T1WI(AxCxS), mm	Pathologic procedure	Primary cancer
1	57F	2.4	Homogeneous	X	O	5x4x4	TR	Lung, adenocarcinoma
2	66/M	3.4	Heterogeneous	O	O	7x7x6	TR	Lung, large cell NEC
3	34/M	3.9	Heterogeneous	O	O	5x5x5	TR	Lung, adenocarcinoma
4	64/F	4.5	Homogeneous	O	X	5x5x2	TR	Lung, adenocarcinoma
5	70/M	2.3	Homogeneous	X	O	4x4x1.5	Supratentorial TR	Lung, adenocarcinoma
6	54/F	4.1	Heterogeneous	O	O	5.5x5.5x5.5	TR	Colon, adenocarcinoma
7	49/F	2.6	Heterogeneous	O	O	4x4x4	TR	Breast, IDC
8	79/F	4.2	Heterogeneous	O	O	4x4x1.5	TR	Lung, adenocarcinoma
9	45/M	2.7	Homogeneous	O	O	7x7x7	Supratentorial TR	Lung, adenocarcinoma
10	40/F	2.3	Heterogeneous	X	O	4x4x4	TR	Breast, IDC
11	39/F	1.3	Homogeneous	O	O	5x5x5	Supratentorial TR	Lung, adenocarcinoma
12	76/M	2.6	Heterogeneous	O	O	5x5x5	TR	Gastric adenocarcinoma
13	50/M	3.6	Heterogeneous	O	X	4x4x1.5	TR	Lung, adenocarcinoma
14	63/F	2.7	Homogeneous	X	O	4x4x4	Supratentorial TR	Breast, IDC
15	45/M	3.5	Homogeneous	O	O	4x4x3	TR	Hepatocellular carcinoma
16	69/M	2.8	Heterogeneous	X	O	7x7x6	TR	Rectal adenocarcinoma
17	77/F	3	Heterogeneous	O	O	4x4x3	TR	Rectal adenocarcinoma
18	43/F	3.5	Heterogeneous	O	O	4x4x1.5	TR	Breast, IDC
19	56/M	2.7	Heterogeneous	O	O	4x4x1	Supratentorial TR	Esophageal small cell NEC
20	65/F	2.4	Heterogeneous	O	O	6x6x6	TR	Breast, medullary carcinoma
21	62/F	2.4	Heterogeneous	O	O	4x4x3	Supratentorial TR	Lung, adenocarcinoma
22	68/M	2.5	Heterogeneous	X	O	5x5x1.5	TR	Lung, sarcomatoid carcinoma
23	56/M	4	Heterogeneous	O	O	4x4x1	TR	Lung, small cell carcinoma
24	66/M	5.5	Heterogeneous	O	O	4x4x1	TR	Gastric adenocarcinoma
25	67/M	5.5	Heterogeneous	O	O	1x1x1	TR	Gastric adenocarcinoma
26	63/F	1.9	Heterogeneous	X	O	4x4x1	Supratentorial TR	Breast, NA

27	55F	3.5	Heterogeneous	O	O	4x4x1	TR	Breast, IDC
28	51/M	4.7	Heterogeneous	X	O	4x4x1	TR	Gastric adenocarcinoma
29	41/F	6.5	Heterogeneous	O	O	4x4x1	TR	Breast, NA
30	45/F	3.6	Heterogeneous	X	X	1x1x1	TR	Lung, adenocarcinoma
31	66/M	3.1	Heterogeneous	O	O	3x3x3	TR	Lung, sarcomatoid carcinoma
32	80/M	4	Heterogeneous	O	O	1x1x0.9	TR	Gastric adenocarcinoma

A, Axial; C, Coronal; S, Sagittal; TR, Tumor removal; NEC, Neuroendocrine carcinoma; IDC, Invasive ductal carcinoma
